# Beyond Glucose: A Brain Energy Bottleneck Hypothesis for Multi‐Energy Substrates in Hypoglycaemia Rescue

**DOI:** 10.1111/dom.70632

**Published:** 2026-03-16

**Authors:** D. Russell‐Jones, M. Laffan, J. Mader

**Affiliations:** ^1^ Royal Surrey NHS Foundation Trust Guildford UK; ^2^ University of Surrey Guildford UK; ^3^ Department of Immunology and Inflammation Imperial College London London UK; ^4^ Division of Endocrinology and Diabetology Medical University Graz Graz Austria

**Keywords:** clinical physiology, diabetes complications, glycaemic control, hypoglycaemia, type 1 diabetes

1

**Plain Language Summary**

- What is the context and purpose of this article?
  ○ Low blood sugar, known as hypoglycaemia, can leave people feeling “foggy” or slow to think, even after blood glucose levels return to normal. For many people with insulin‐treated diabetes, these episodes occur frequently and can affect daily functioning, confidence and quality of life. Studies show that cognitive function (thinking, attention, and reaction time) often remains impaired for some time after blood glucose has been corrected. This suggests that restoring glucose in the bloodstream does not immediately restore energy inside brain cells.
  ○ This article proposes a new explanation for this delay, described as a “brain energy bottleneck.” It examines whether multi‐energy substrates for hypoglycaemia (MESH) treatments, which combine glucose with additional energy sources such as beta‐hydroxybutyrate (BHB) and lactate, including the novel product FLO23011 (Klario), may help improve recovery after low blood sugar.
- What is the proposed mechanism?
  ○ The proposed mechanism centres on a molecule called NAD^+^, which brain cells need in order to convert glucose into usable energy. During hypoglycaemia, stress within brain cells may activate an enzyme called PARP‐1, which consumes NAD^+^, reducing its availability. When NAD^+^ levels fall, an important step in converting glucose into energy becomes less efficient. As a result, the brain may temporarily struggle to use glucose properly, even after blood glucose has been corrected.
  ○ Alternative energy sources such as BHB and lactate can enter the brain's energy‐producing system through different pathways that do not rely on the blocked step in glucose breakdown. Providing these substrates alongside glucose may therefore help sustain energy production while normal glucose metabolism recovers.
- What evidence supports this idea?
  ○ Studies in brain cells show that glucose deprivation can trigger stress responses, including activation of PARP‐1, which may reduce NAD^+^ levels and temporarily impair the cell's ability to turn glucose into energy. Animal studies suggest that giving alternative energy sources with glucose after hypoglycaemia can help support the brain's energy supply, reduce brain cell injury, and preserve memory and learning.
  ○ Human studies have shown that raising levels of BHB or lactate alongside glucose during hypoglycaemia can lessen symptoms and speed up cognitive recovery. In the first clinical study in people self‐managing hypoglycaemia, more than 1,000 episodes were analysed to evaluate a MESH product, FLO23011, combining glucose with BHB and lactate. Use of the formulation was linked to fewer repeat episodes and better patient‐reported recovery compared with glucose alone. These findings do not directly confirm the mechanism in people, but they are consistent with this proposed explanation.
- Why is this important?
  ○ Drawing on evidence from experimental and clinical research, this article offers a new way of understanding why cognitive recovery can lag behind the return of normal blood sugar levels. The brain energy bottleneck hypothesis provides a biologically plausible explanation for the recovery benefits seen in recent studies of MESH treatments such as FLO23011 and may inform the evolution of hypoglycaemia self‐care beyond glucose alone.

This commentary proposes a brain energy bottleneck as a mechanistic hypothesis that could help explain observed benefits of combining glucose with alternative oxidative substrates in hypoglycaemia. Multi‐energy substrates for hypoglycaemia (MESH) refer to formulations that combine glucose with alternative oxidative substrates such as beta‐hydroxybutyrate (BHB) and lactate. In vitro data, preclinical studies in animals, and small clinical studies in humans suggest these combinations may improve cognitive recovery and resolution of neuroglycopenic symptoms after hypoglycaemia compared with glucose alone.

We propose that delayed cerebral energy recovery following hypoglycaemia may result from nicotinamide adenine dinucleotide (NAD^+^) depletion during hypoglycaemia, inducing a transient glycolytic bottleneck that persists after plasma glucose restoration and constrains neuronal glucose utilisation. Accordingly, post‐hypoglycaemia cerebral energy failure reflects more than a transient lack of glucose; convergent experimental evidence indicates that poly(ADP‐ribose) polymerase (PARP‐1) activation after glucose correction and associated NAD^+^ consumption can transiently limit glycolytic capacity, thereby constraining neuronal glucose utilisation after plasma glucose correction (Figure 1) [1, 2, 3, 4].

**FIGURE 1:**
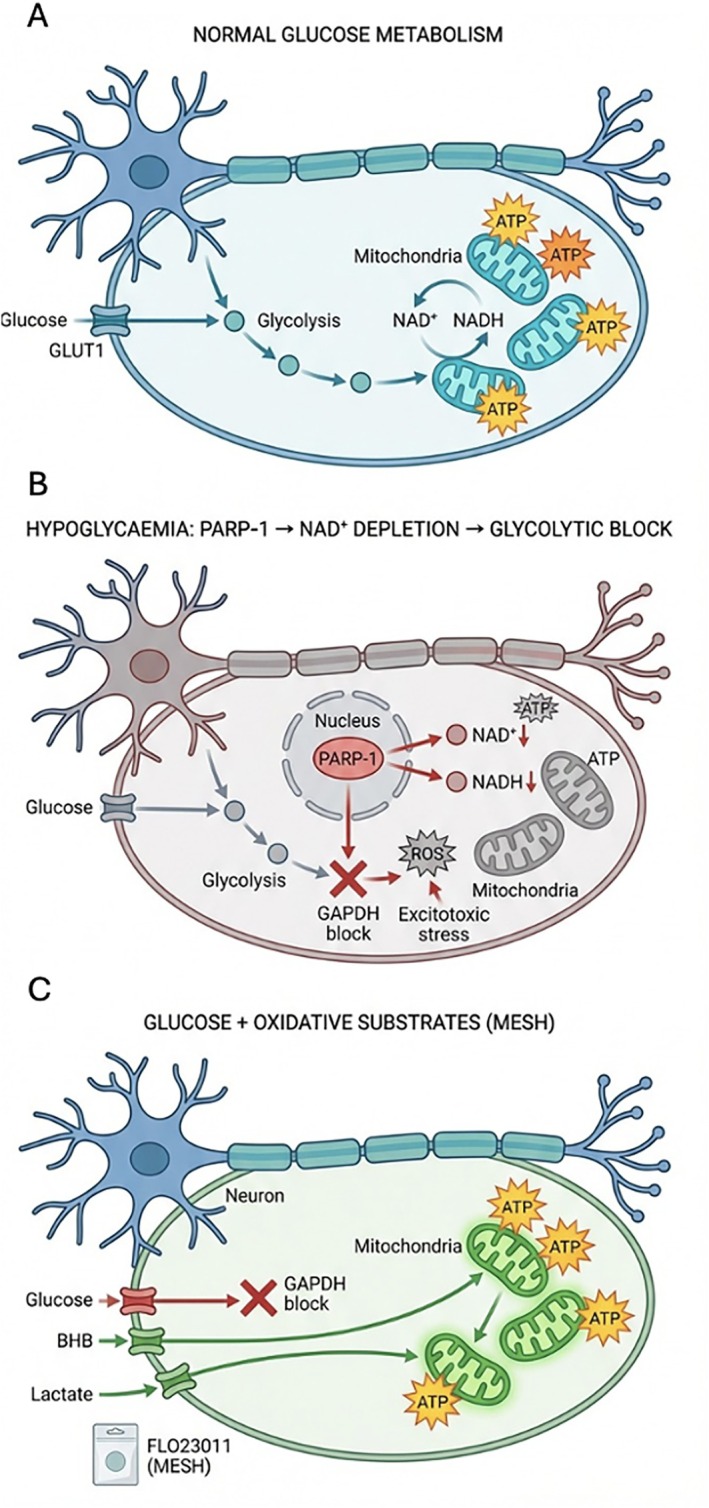
Mechanistic model of the proposed Poly [ADP‐ribose] polymerase 1 (PARP‐1)/Nicotinamide adenine dinucleotide (NAD^+^) bottleneck hypothesis. (a) Normal energy metabolism. Glucose is the principal neuronal substrate, metabolised via glycolysis to pyruvate, which enters mitochondria for complete oxidation via the tricarboxylic acid (TCA) cycle, generating ATP. (b) Proposed energy bottleneck during hypoglycaemia. Experimental findings: In cellular and animal models of severe hypoglycaemia and energy stress, PARP‐1 activation is associated with NAD^+^ consumption. NAD^+^ depletion under these conditions has been shown to impair NAD^+^‐dependent steps of glycolysis and reduce glycolytic flux. Hypothetical extension: We propose that this NAD^+^ depletion creates a bottleneck at glyceraldehyde‐3‐phosphate dehydrogenase (GAPDH), impairing glycolytic flux and delaying ATP recovery even after plasma glucose normalisation. (c) Proposed therapeutic strategy (hypothesis‐generating). Addition of alternative oxidative substrates (beta‐hydroxybutyrate [BHB], lactate) which enter mitochondrial metabolism downstream of glycolysis, may help sustain ATP production during the recovery window if glycolytic capacity is transiently constrained. Translational and early clinical studies suggest that these substrates can attenuate neuroglycopenic symptoms and support cognitive recovery during hypoglycaemia. However, confirmation that this benefit operates via the proposed NAD^+^‐dependent bottleneck mechanism in humans remains to be established.

The brain has high energy demands but minimal energy storage, requiring continuous substrate supply to maintain neuronal activity and predominantly relies on glucose for this purpose. Alternative energy sources, such as ketone bodies (BHB, acetoacetate), lactate and pyruvate, play greater roles in cerebral metabolism during starvation and exercise [5]. This physiological capacity and the growing interest in using BHB as a sport performance supplement provide the foundation for hypothesising that exogenous provision of alternative energy sources may support recovery from hypoglycaemia‐induced neurological impairment.

We propose that these energy sources may improve hypoglycaemia recovery: BHB and lactate enter oxidative metabolism downstream of the glycolytic bottleneck thereby helping sustain mitochondrial ATP production when glycolytic capacity is constrained [6]. In experimental models of hypoglycaemia and cerebral energy stress, provision of these energy substrates has been associated with preservation of neuronal energy status and reduced neuronal injury [1, 7, 8, 9], supporting the functional relevance of this metabolic bypass, although direct demonstration of this pathway in human hypoglycaemia is currently lacking.

Within this framework, glucose combined with alternative oxidative substrates represents a plausible approach for supporting cerebral energy recovery following hypoglycaemia, with potential importance for cognitive recovery. This concept is informed by experimental evidence and is illustrated by data from MESH formulations, including a glucose‐BHB‐lactate combination, FL023011 [10]. This may be the starting point for work to advance hypoglycaemia management from ‘glucose alone’ to ‘glucose plus an oxidative substrate’.

## 2 Clinical Problem and Unmet Need

Hypoglycaemia is a common problem in insulin‐treated diabetes, with many experiencing 100–200 episodes/year, impacting quality of life [11]. Guidelines recommend 15–20 g rapidly absorbed carbohydrate to restore plasma glucose levels. Hyperinsulinemia clamp studies in adults with Type 1 diabetes show cognitive performance on choice reasoning tasks can remain impaired for up to 40 min after euglycemia is restored [12]. Electrophysiological studies in insulin‐dependent diabetes and clamp studies in healthy volunteers show cognitive recovery lags symptom resolution and normalisation of plasma glucose by 45–75 min [13, 14].

## 3 Mechanistic Hypothesis: The Energy Bottleneck

We propose a mechanistic model whereby activation of PARP‐1 can deplete cytosolic NAD^+^, inhibit glyceraldehyde‐3‐phosphate dehydrogenase (GAPDH), and thereby impair glycolytic flux.

Experimental studies in cultured rodent neurons demonstrate that even moderate glucose deprivation is sufficient to increase susceptibility to glutamate‐mediated excitotoxicity [15]. Under conditions of energy deficit, glutamate excitotoxicity has been linked to zinc release, reactive oxygen species (ROS) generation and activation of PARP‐1 which depletes NAD^+1–3^. This cascade has been proposed to impair glycolytic flux, plausibly through inhibition of NAD^+^‐dependent steps such as GAPDH, limiting neuronal glucose utilisation even after systemic glucose levels are restored [4]. PARP‐1‐mediated NAD^+^ depletion and the resulting GAPDH bottleneck are therefore framed as a plausible contributor to delayed cerebral energy recovery in humans.

Experimental models support a PARP‐1–dependent impact on NAD^+^ availability and glycolytic flux; however, the temporal dynamics and magnitude of PARP‐1 activation, NAD^+^ depletion and downstream GAPDH modulation during self‐treated hypoglycaemia in humans remain undefined. This pathway is presented as a biologically plausible framework that may contribute to neuroglycopenic symptomatology, and as a suggested mechanism that might be shown to operate in the day‐to‐day hypoglycaemia experience of people with diabetes.

NAD^+^ pools are compartmentalised within the cell and PARP‐1‐mediated depletion predominantly affects cytosolic NAD^+16^. As a result, cytosolic glycolysis may be impaired, while mitochondrial oxidative pathways remain preserved. Glucose metabolism requires four NAD^+^ → NADH conversions: two of which are cytosolic (GAPDH) and two are mitochondrial (pyruvate dehydrogenase); cytosolic NADH potential must be shuttled to mitochondria. In experimental glucose deprivation, depletion of cytosolic NAD^+^ has been shown to inhibit GAPDH, creating a glycolytic bottleneck that reduces glycolytic flux and leads to reduced ATP availability, an effect reversible by NAD^+^ repletion or provision of downstream oxidative substrates [16].

In contrast, alternative energy sources, such as BHB and lactate, enter cerebral metabolism downstream of glycolysis. BHB is oxidised within mitochondria (BHB → acetoacetate → acetoacetyl‐CoA → 2 acetyl‐CoA). Lactate conversion to pyruvate involves NAD^+^‐dependent steps primarily in the cytosol, but pyruvate is removed to enter the TCA cycle, and these substrates, in experimental systems, support mitochondrial ATP under conditions of reduced glucose flux [6]. These metabolic pathway considerations provide a basis to explain why neuronal recovery may lag systemic glycaemic correction in practice.

Together, these findings reframe the potential effects of glucose reintroduction. Rather than uniformly marking recovery, glucose reperfusion is associated with secondary oxidative stress in both neuronal culture and in vivo models. In experimental systems, this can be injurious: restored glucose can support metabolic pathways that increase oxidative stress, including flux through the hexose monophosphate pathway, which increases NADPH and drives a secondary burst of superoxide via NADPH oxidase [17]. This oxidative surge has been shown to cause neuronal loss, particularly in vulnerable regions such as the hippocampal CA1 in animal models.

Identification of this glycolytic block provides a rationale for using alternative energy sources as oxidative substrates, such as BHB, to sustain mitochondrial ATP production during the recovery window and support neuronal function until glycolytic competence is restored. These observations derive from cell and animal models and may not fully extrapolate to human hypoglycaemia, where the magnitude and timing of PARP‐1 activation and NAD^+^ depletion are likely to vary between severe, non‐severe and recurrent episodes. The ‘brain energy bottleneck’ is presented as a mechanistic hypothesis that may explain the dissociation between systemic glycaemic correction and delayed cognitive recovery.

Animal models provide evidence supporting the hypothesis of a brain energy bottleneck and the ability of alternative energy sources alongside glucose to improve post‐hypoglycaemia recovery. In a rat model of insulin‐induced severe hypoglycaemia, administering pyruvate with glucose after the insult was associated with ~70%–90% reductions in neuronal death in hippocampal and cortical regions, along with preservation of long‐term cognitive performance [1]. These findings are consistent with the idea of NAD^+^‐independent entry of pyruvate into the tricarboxylic acid cycle (TCA), bypassing metabolic constraints arising from hypoglycaemia, including those linked to PARP‐1 activation.

Related experimental work in non‐severe and recurrent/moderate hypoglycaemia is potentially more representative of routine clinical experience: In rodent models, repeated post‐hypoglycaemic administration of pyruvate with glucose was associated with the attenuation of cortical neuronal injury, zinc accumulation, oxidative injury, microglial activation, PARP‐1 activation and glutathione loss [7]. Complementary studies show that lactate given with glucose can reduce hippocampal neuronal loss by ~80% in vivo [8]. Such studies demonstrate the potential of alternative energy sources to sustain oxidative metabolism during the period in which glucose utilisation may be compromised.

BHB extends this paradigm: In a rat model of non‐coma hypoglycaemia, systemic BHB administration during and after hypoglycaemia preserved cortical ATP levels, reduced ROS, and prevented cortical neuronal death [9]. BHB may also modulate oxidative stress, inhibit NLRP3 inflammasome activity and HCA2‐dependent signalling, supporting neuronal resilience beyond its role as an energy substrate [18]. Its protective effects may reflect both sustained energy provision and attenuation of oxidative stress. Although direct involvement of the PARP‐1‐NAD^+^ pathway in humans has not been established, such effects support the hypothesis.

## 4 Translational Implications

Human insulin clamp studies show that BHB and lactate can buffer the impact of hypoglycaemia: symptoms are attenuated, and cognitive recovery (attention, reaction time) occurs more rapidly when these substrates are elevated [19]. This supports the view that exogenous BHB, or lactate can sustain cortical function when glucose availability and/or glycolytic capacity are constrained, although these conditions may not fully reflect real‐world diabetes care. Nonetheless, in adults with type 1 diabetes, oral medium‐chain triglycerides that raise BHB levels protect cognition during hypoglycaemia, attenuating memory decline and improving processing speed [20].

Recently, a MESH formulation combining glucose, BHB and lactate (FLO23011) was evaluated in pragmatic clinical studies [10]. In a randomised, open‐label, crossover trial in 12 adults with Type 1 diabetes, the formulation was compared with standard oral glucose across more than 1000 real‐world hypoglycaemic episodes. The MESH formulation was associated with higher Continuous Glucose Monitoring (CGM) time‐in‐range, 27% fewer recurrent hypoglycaemic episodes in the hours after treatment, and patient‐reported improvements in the resolution of neuroglycopenic symptoms and fatigue. These small, open‐label data are hypothesis‐generating; they do not provide mechanistic validation but illustrate how the proposed brain energy bottleneck might have translational relevance. Although the episode‐level dataset of > 1000 episodes is extensive, a study at this scale is optimised to detect within‐participant differences in glycaemic trajectories and symptoms rather than to provide definitive estimates of rare safety or longer term outcomes. The open‐label structure was chosen to reflect real‐world self‐treatment conditions and to ensure feasibility of repeated hypoglycaemia treatment over 12 weeks; nevertheless, this design can introduce expectancy and reporting biases for subjective symptom endpoints. CGM endpoints are influenced by behaviours such as insulin adjustment, carbohydrate intake and physical activity; accordingly, CGM‐derived outcomes (time in range, recurrent hypoglycaemia) are interpreted in the context of the crossover design and consistent participant‐level exposure to both treatments rather than as stand‐alone population benchmarks.

## 5 Conclusions

This hypothesis proposes the therapeutic principle of combining rapid glucose delivery with alternative oxidative substrates such as BHB and lactate in appropriate hypoglycaemia settings, to support cerebral energy recovery when glycolytic capacity may be transiently constrained by a brain energy bottleneck. Building on convergent in vitro, animal, and clinical data, this framework offers a rationale that may inform the evolution of hypoglycaemia management from ‘glucose alone’ towards ‘glucose plus an oxidative substrate’ where appropriate in day‐to‐day care and provides a focused agenda for mechanistic, translational and formulation research to refine its application.

## Funding

The authors have nothing to report.

## Conflicts of Interest

D. Russell‐Jones will be named on a patent for FLO2311/Klario if this is granted. He has not received any payment for work on Klario by Flow Health Science Inc. He was the PI on the clinical studies of FLO23011; he did not receive payment for this work. He receives research funding or advisory board honoraria from Abbott Diabetes Care, Dexcom, Astra Zeneca, Eli Lilly, Medtronic, Novartis, Novo Nordisk A/S and Sanofi‐Aventis. J. Mader is a member of advisory boards of Abbott Diabetes Care, Becton‐Dickinson, Biomea Fusion, Dexcom, Eli Lilly, Embecta, Medtronic, myLife, Novo Nordisk A/S, Pharmasens, Roche Diabetes Care, Sanofi‐Aventis, Tandem, Viatris and received speaker honoraria from A. Menarini Diagnostics, Abbott Diabetes Care, Dexcom, Eli Lilly, Medtrust, MSD, Novo Nordisk A/S, Roche Diabetes Care, Sanofi, Viatris and Ypsomed. She is a shareholder of decide Clinical Software GmbH and elyte Diagnostics and serves as CMO of elyte Diagnostics. J. Mader has not received any payment for work on FLO23011/Klario by Flow Health Science Inc. M. Laffan has not received any payment for work on FLO23011/Klario by Flow Health Science Inc.

## Data Availability

Authors agree to make data and materials supporting the results available upon reasonable request.
